# The potential of urban trees to reduce heat-related mortality in London

**DOI:** 10.1088/1748-9326/ad3a7e

**Published:** 2024-04-12

**Authors:** Jonathon Taylor, Charles Simpson, Oscar Brousse, Anna-Kaisa Viitanen, Clare Heaviside

**Affiliations:** 1 Department of Civil Engineering, Tampere University, Tampere, Finland; 2 UCL Institute for Environmental Design and Engineering, UCL, London, United Kingdom

**Keywords:** urban heat island, personal weather stations, climate change, heat mortality, tree canopy

## Abstract

Increasing temperatures and more frequent heatwave events pose threats to population health, particularly in urban environments due to the urban heat island (UHI) effect. Greening, in particular planting trees, is widely discussed as a means of reducing heat exposure and associated mortality in cities. This study aims to use data from personal weather stations (PWS) across the Greater London Authority to understand how urban temperatures vary according to tree canopy coverage and estimate the heat-health impacts of London’s urban trees. Data from Netatmo PWS from 2015–2022 were cleaned, combined with official Met Office temperatures, and spatially linked to tree canopy coverage and built environment data. A generalized additive model was used to predict daily average urban temperatures under different tree canopy coverage scenarios for historical and projected future summers, and subsequent health impacts estimated. Results show areas of London with higher canopy coverage have lower urban temperatures, with average maximum daytime temperatures 0.8 °C and minimum temperatures 2.0 °C lower in the top decile versus bottom decile canopy coverage during the 2022 heatwaves. We estimate that London’s urban forest helped avoid 153 heat attributable deaths from 2015–2022 (including 16 excess deaths during the 2022 heatwaves), representing around 16% of UHI-related mortality. Increasing tree coverage 10% in-line with the London strategy would have reduced UHI-related mortality by a further 10%, while a maximal tree coverage would have reduced it 55%. By 2061–2080, under RCP8.5, we estimate that London’s current tree planting strategy can help avoid an additional 23 heat-attributable deaths a year, with maximal coverage increasing this to 131. Substantial benefits would also be seen for carbon storage and sequestration. Results of this study support increasing urban tree coverage as part of a wider public health effort to mitigate high urban temperatures.

## 1. Introduction

Climate change is leading to elevated global average temperatures and increased frequency and severity of heatwave events [1]. London is already experiencing extreme heat (EH) episodes, for example in 2022 when heatwave temperatures exceeded a record 40 °C, an event estimated 160 times more likely because of climate change [2]. Heat exposure may be exacerbated by the urban heat island (UHI) effect, where urban centres are typically hotter than surrounding rural areas. UHIs are primarily caused by human modification of land surfaces, replacing natural vegetation with materials like concrete and asphalt that have different thermal and surface radiative properties and which absorb and retain heat during the day [3]. In London, the difference between the urban monthly mean maximum urban and surrounding rural temperature has been observed around 1.4 °C–2.9 °C [4, 5], with lower intensities found in more vegetated areas.

Heat exposure can lead to several negative physical health effects, with older people, the socially isolated, very young children, and those with chronic illnesses the most vulnerable [6]. It can lead to cardiovascular strain, respiratory distress, dehydration, and heat stroke, and exposure to excess heat is associated with all-cause and cardiorespiratory mortality, adverse pregnancy outcomes, and mental health problems [1, 6, 7]. During the 2022 heatwaves, 2985 all-cause excess deaths were observed in England, 387 of which were in London [8]. While rates of heat-attributable deaths are greatest during extreme weather, many deaths occur during the more frequent warm and hot days [9].

Therefore, focus on urban heat mitigation and climate adaptation is growing. Increasing vegetation is one way to decrease near-surface air temperatures [3, 10] due to changes in surface material properties, shading, and evapotranspiration, where plants absorb water and release it as water vapour. Trees, in particular, have been widely studied to assess their potential for mitigating heat in urban environments [11–15] because their leaves and branches provide shading and they have higher transpiration rates than low vegetation. However, the relationship is complex, as increased surface roughness from trees can reduce windflow and heat dissipation [14], and recent evidence suggests that in many European cities, trees have a small or negligible effect on daytime air temperature [15]. Estimating any heat-health benefits provided by trees can be complicated due to meteorological data scarcity in urban environments, meaning many studies rely on remotely-sensed land surface temperature (LST). A recent study using LST showed that 25% of UHI-attributable deaths could have been avoided in 2015 if tree coverage was increased by 30% in London [16]. However, LST can be a poor predictor of urban-canopy air-temperature [15, 17, 18], and further work is required to disentangle how trees are associated with higher or lower heat-related mortality locally.

The recent growth of personal weather stations (PWS) has provided opportunities to use crowd- sourced meteorological data in urban climate studies [19–23] that have typically relied on limited numbers of official meteorological stations, researcher-measured conditions, or climate models. PWS popularity has led to increased density of PWS networks in cities and improved geographical coverage of monitored data. While PWS have greater uncertainty than official monitoring stations and data may be noisy [24], various methods have been developed to perform quality control on data [25]. A recent study used PWS to examine the cooling efficiencies of tree cover in European cities, finding that trees have a smaller impact on air than surface temperatures, and may not have a cooling effect in all cities [15]. Studies have used PWS, for example, to examine the impacts of land cover on temperatures [5, 15, 26–28], model air temperatures [29–31], as an input to building physics models [32], and to validate and bias-correct urban climate simulations [24, 33].

This paper uses the dense network of PWS across the Greater London Authority (GLA) to evaluate differences in air temperature according to tree canopy cover, and uses this association to estimate the potential for trees to reduce heat exposure and associated mortality under historical and future climate scenarios. To do this, we derive a large dataset of PWS and official station air temperature data from October 2015–September 2022, inclusive. We then spatially link PWS temperatures to tree canopy cover and building coverage (BC) and height data for London and temporally to temperatures at Heathrow Airport. From this, a generalized additive model (GAM) is developed and used to predict daily average urban temperatures under different tree canopy coverage scenarios with health impact calculations used to estimate how changes to tree canopy coverage may change heat-related deaths.

## 2. Methods

### 2.1. Temperature data

Near-surface temperate data was obtained from two different sources from 01 October 2015–30 September 2022: official Met Office weather stations and Netatmo PWS stations within a wide bounding domain that includes the GLA. This timeframe includes the historically hottest summer of 2018, and the three exceptional heatwaves that occurred during the summer of 2022 (15th–17th June, 17th–19th July, and 9th–15th August) when temperatures reached a maximum of 39 °C at London Heathrow.

Hourly temperatures, station ID, and coordinates for Met Office stations were obtained from September 2015–December 2022 from MIDAS Open [34]. This included the station at London Heathrow, commonly used for studying the London climate and which offers a comprehensive coverage of the period of interest. This was used as a reference station.

PWS station ID, and coordinates was obtained using the *Patatmo* Python module that utilizes the Netatmo API (https://dev.netatmo.com/). This resulted in 619 stations, of which 502 were within GLA administrative boundaries. Hourly air temperatures were then downloaded for PWS stations for the study period. Cleaning and analysis of PWS temperatures was carried out using *R*. First, the altitude of each station was spatially joined using a 10 m resolution Digital Elevation Model of London [35]. Height correction and outliers removal was performed using CrowdQC+ [25], a tool developed for quality control of PWS weather data. Hourly measurements were removed if they were taken by duplicate stations located at the same coordinates (Step M1); were outliers, as determined by their *z*-score (Step M2); if more than 20% of the measurements in the month were removed in the previous steps, indicating the PWS may be unreliable (Step M3); and finally if the measurements were not correlated to the median temperature of other measurements (Pearson *r* < 0.9) and are assumed to be indoors (Step M4). We then excluded days with more than three hours of data absent. This reduced the number of stations within the GLA boundaries to 490, with 520 623 sensor-days of valid PWS measurements.

**Figure 1.**
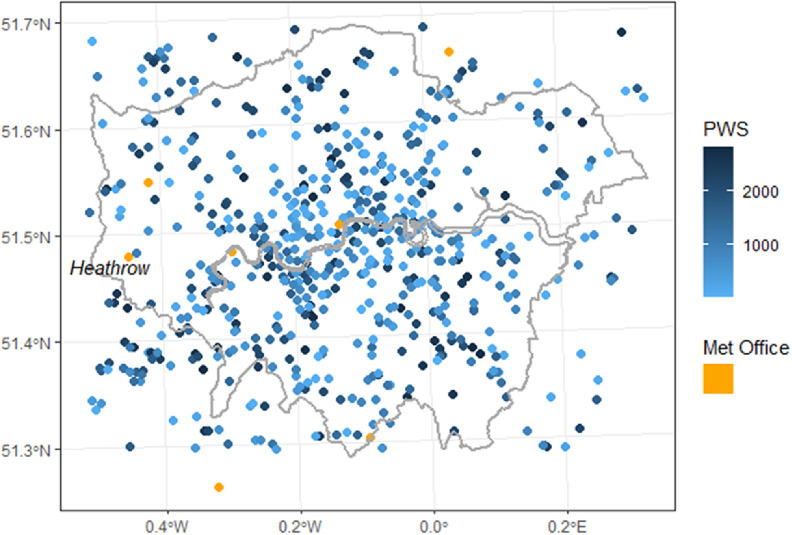
Netatmo stations, by days of measured data, and Met Office stations within the GLA administrative boundaries and wider domain.

The Met Office and PWS temperatures were combined into a common dataset (figure 1), except for Heathrow which was used as a reference temperature for each date. Stations located in rural areas outside the GLA were used as a baseline to estimate UHI intensity. These rural areas were identified via a spatial join to 2011 Office for National Statistics rural/urban classifications [36] for different census output areas.

#### 2.1.1. Environmental and population data

**Figure 2.**
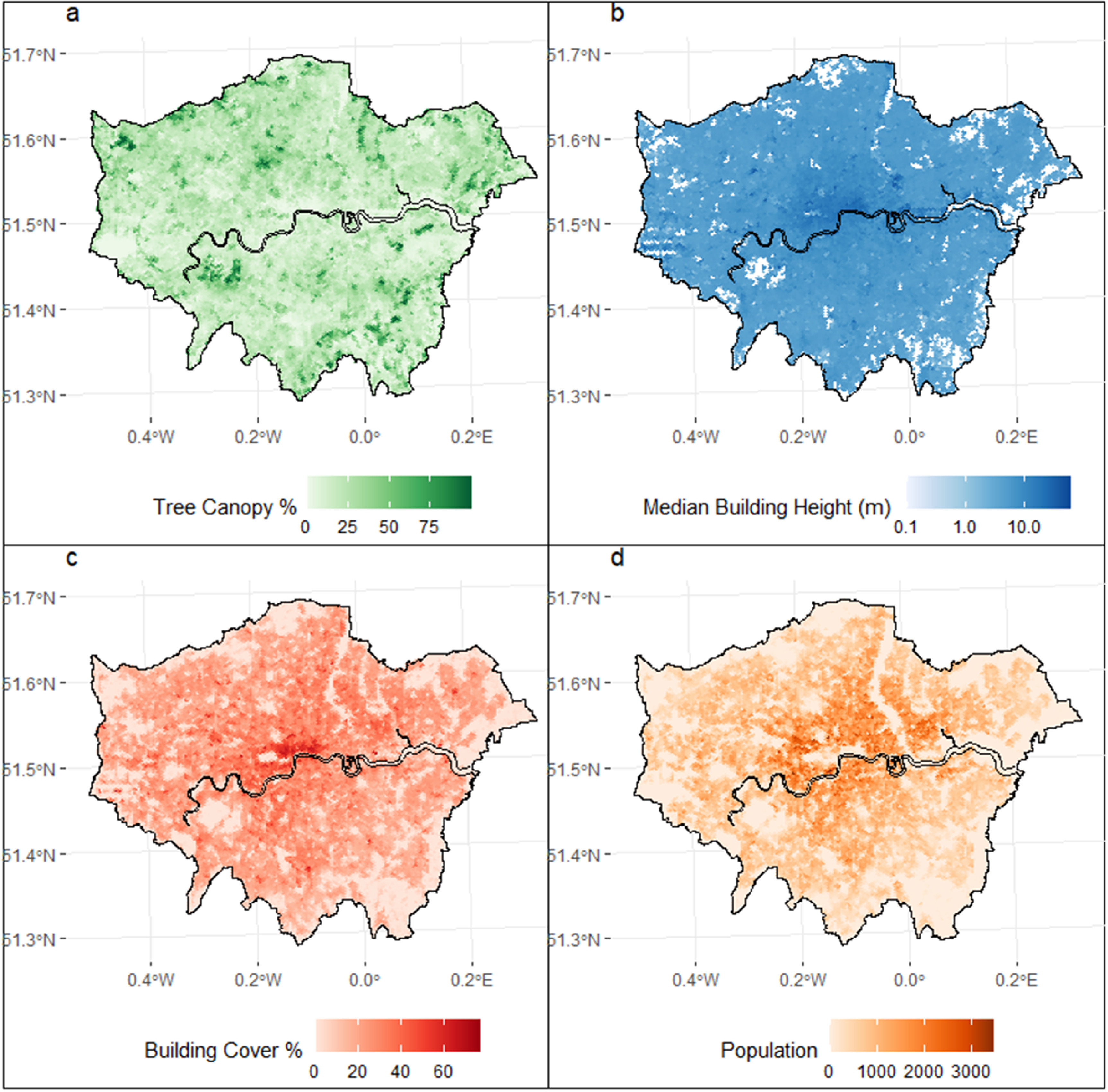
For each hexagon, (a) percent tree canopy coverage (b) median building height, and (c) percent coverage of buildings, and (d) population density.

Tree canopy coverage data for the GLA is from the London Data Store [37] (figure 2(a)). The dataset contains 15 041 hexagons, each 350 m across, derived from high resolution (10 cm per pixel) colour infrared imagery collected in September 2016, with pixels as being either a tree canopy or not using a machine learning algorithm. Further details on how the tree canopy data was derived can be found in [38].

Urban temperatures also vary according to local climate zones (LCZs), or local surface structure and cover. As tree canopy is assumed to be correlated with the natural component of LCZs, we derived two continuous variables to describe the height and cover of the built component. For each hexagon, a spatial join to the OS MasterMap Building Height Attribute [39] dataset was used to calculate the median height of buildings (removing buildings with low measurement confidence) (figure 2(b)) and the percent area covered by all buildings (figure 2(c)). OpenPopGrid [40], a 10 m gridded population dataset, was used to calculate the population within each hexagon (figure 2(d)). To account for broader spatial impacts, buffers around the hexagons at 200, 500, and 1000 m were drawn and the area-weighted average BC and building height (BH) re-calculated within these buffers.

Hexagon canopy coverage was then adjusted for different scenarios:

a) *Current scenario*: a base case with existing tree canopy coverage.
b) *Grey scenario*: tree canopy cover is reduced to near zero, representing a scenario where trees are reduced to the lowest possible amounts seen per BC. In this scenario, the tree coverage was set to 0.05th percentile for each hexagon.
c) *London strategy:* tree canopy coverage each hexagon is increased 10%, as per the London Environment Strategy goals for 2050 [41].
d) *Average strategy*: hexagons below the population-weighted average canopy coverage are increased to the average.
e) *Green scenario*: tree canopy cover is increased to an amount that represents a maximum amount, constrained by existing BC. Quantile regression was used to identify the maximum (99.5th percentile) tree coverage according to the BC within each hexagon. In cases where tree coverage exceeded the 99.5th percentile, the current coverage was maintained.

Finally, to estimate the carbon benefits of these scenarios, the estimated 2.4 million tonnes of carbon stored and 77 200 tonnes of carbon sequestered annually by London’s trees [42] were recalculated given the proportional changes in tree coverage.

### 2.2. Analysis and modelling

Analyses were performed to produce descriptive statistics on UHI intensity and examine how air temperatures vary by the amount of tree canopy coverage.

Hourly temperature data was used to calculate UHI intensity for each day, defined as the maximum hourly difference between stations within the GLA boundaries (90th percentile) and the average of stations in the larger domain that are classified as rural. For the three heatwaves during the 2022 summer, the UHI intensity, hourly timeseries, and differences in 24 h, daytime, and nighttime mean, maximum, and minimum temperatures in areas with differing amounts of tree coverage were calculated. Daily (*d*) 24 h mean (${T_{{\text{mean}},\;d}}$) was calculated for all stations and joined by date to the corresponding value at the heathrow reference station (${T_{{\text{mean}},\;d,\;{\text{heathrow}}}}$).

GAMs were then fit to the aggregated daily data using the *mgcv* package in *R*. GAMs were used because they allow the response variable to depend on smooth functions of the predictor variables; this can present an advantage over linear regression models which contain the assumption that response is linear in the predictor variables. Another advantage is the assumption that effects of each variable are separate, enabling extrapolation to the counterfactual scenarios. The effects of the predictor variables were assumed to be independent to enable counterfactual reasoning about the effect of changing tree cover while keeping other aspects of the built environment the same. Smoothing parameters were estimated using restricted maximum likelihood (REML), with degrees of freedom limited to 9 *a priori* to improve transferability for the counterfactual scenario and interpolation between data points. The response variable was ${T_{{\,\text{mean}},\;d}}$ and the predictor variables were ${T_{{\,\text{mean}},\;d,\;{\text{heathrow}}}}$; tree canopy cover, BC, and BH in the hexagon which the station is located; and the BC and BH within 200 m, 500 m, or 1000 m buffers.

Models were fit for the different buffer sizes, using 5-fold cross-validation (stratified by station) to identify the buffer best able to predict temperatures at an unseen station. Model residuals were inspected, testing for spatial autocorrelation using a variogram and calculation of Morans I (for the whole dataset, summers, and each day), and examined for temporal autocorrelation using the Durban–Watson test.

### 2.3. Heat and health impact modelling

The GAM model was then used to estimate temperature exposures from 2015–2022 under different tree coverage scenarios and the corresponding health impacts calculated. Rural temperatures from 2015–2022 were used as a baseline. For the same time period, the GAMS model was used to predict the daily mean temperature (${T_{{\text{mean}},\;d,k}}$) for each hexagon (*k*) under different canopy coverage scenarios. For each day, the population-weighted mean daily temperature for the GLA was calculated (equation (1)):

\begin{align*}\overline {{T_{{\,\text{mean}},\;d}}} = \frac{{{{\mathop \sum \nolimits}}_{i = 1}^n\left( {{T_{{\text{mean}},\;d,k}}} \right) \times {P_k}}}{{{{\mathop \sum \nolimits}}_{i = 1}^n{P_k}}}\end{align*}

where ${P_k}$ is the population within a hexagon.

Health impact calculations were then carried out to estimate daily heat-related mortality. We use the heat-mortality relationship for London from Arbuthnott *et al* [43] (table 1), which derived a relative risk (RR) of mortality for different age groups (*i*) using lag 0, 1 ${T_{{\text{mean}},\;d}}$ (herein referred to as ${T_{{\text{mean}},\;d,{\text{lag}}}}$) for London. When ${T_{{\text{mean}},\;d,{\text{lag}}}}$ exceeds a temperature threshold (${T_h}$, or 18.9 °C in London) the RR of heat related mortality increases per degree C:

\begin{align*}R{R_{i,d}}&amp; = {e^{{c_a}\left( {{T_{{\text{mean}},\;d,\;{\text{lag}}}} - {T_h}} \right)}}\;{\text{when}}\;{T_{{\text{mean}},\;d,\;{\text{lag}}}} &gt; {T_h} \nonumber\\ R{R_{i,d}}&amp; = 0 \,\,\kern58pt{\text{when}}\;{T_{{\text{mean}},\;d,\;{\text{lag}}}} \unicode{x2A7D} {T_h}\end{align*}

**Table 1.** Heat-mortality relationship for London, from Arbuthnott *et al* [43].

Age group	Threshold, ${T_h}$	RR of mortality (95% CI), total
0–64	18.9 °C	1.022
65–74		1.024
75–		1.049

where *c*(*a*) is the natural log of the RR at 1 °C above the threshold. The attributable fraction (AF) of heat-related mortality is calculated as:

\begin{equation*}A{F_{i,d}} = \frac{{R{R_{i,d}} - 1}}{{R{R_{i,d}}}}.\end{equation*}

This was used alongside the number of deaths per each day of the year for different age groups (${q_{i,d}}$) in London (2015–2022) [44, 45] to calculate heat-related mortality and summed across all days and ages to estimate attributable mortality (AM):

\begin{equation*}AM = \sum\limits_d \sum\limits_i {q_{i,d}} \times A{F_{i,d}}.\end{equation*}

Heat AM under different scenarios was also examined using projected (1980–2010, 2041–2060, 2061–2080) climates for London for RCP8.5 from UKCP18 [46]. This data includes 12 ensemble members of average temperatures for each day of the year for these time periods. The GAM model was used to estimate $\overline {{T_{{\text{s}},d}}} $ for projected climates, and AM estimated using the number of deaths per day of year and age group averaged over 2015–2022.

## 3. Results

### 3.1. Tree coverage

**Figure 3.**
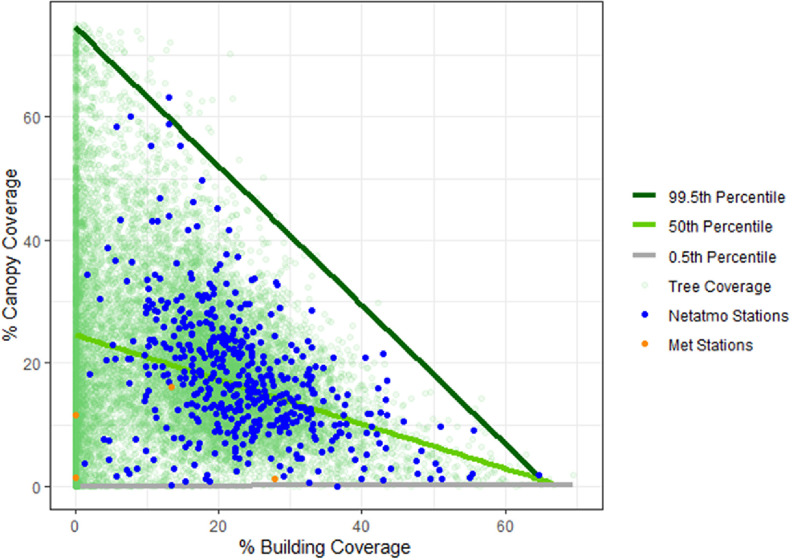
The variation of tree canopy coverage and building density for hexagons (green), PWS stations (blue), and Met Office stations (orange) with quantile regression for the hexagons.

The tree canopy coverage varied significantly, with a population-weighted average of 17.5% (range of 0%–99.9%), and a total of 336 km^2^ of tree canopy with the GLA. The results of the quantile regression showed a correlation between the 99.5th percentile tree canopy and BC (figure 3) (1.5% reduction in tree canopy per 1% increase in building area, *p* = 0.00). PWS stations were relatively representative of the distribution of populated hexagons, although the maximum tree coverage was 64%. Population-weighted tree canopy coverage increased to 58% under the greening scenario (1242 km^2^) and 21% (395 km^2^) under the average scenario. Met stations were in areas with lower canopy coverage.

### 3.2. Temperatures

**Figure 4.**
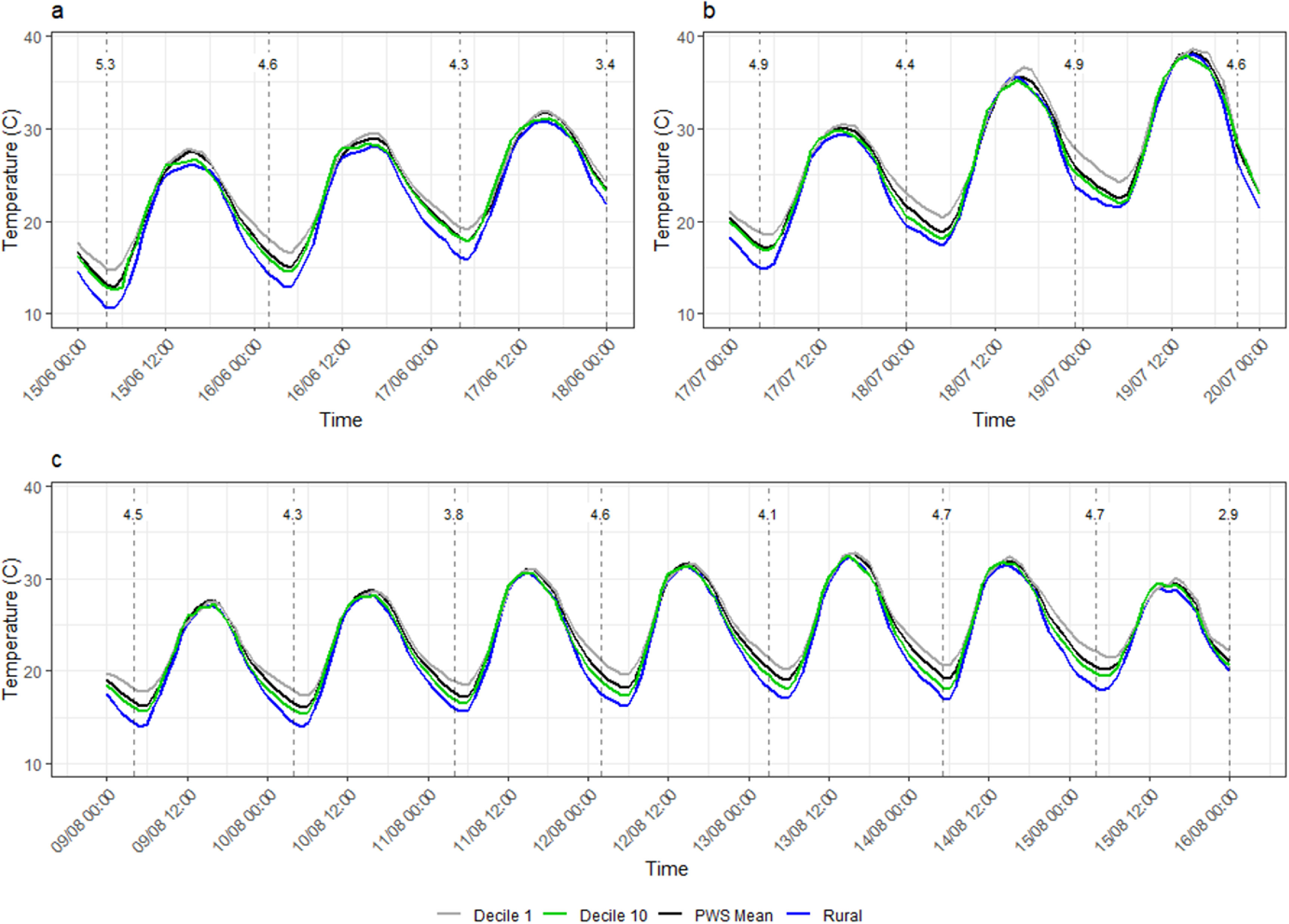
London PWS temperatures during the summer 2022 heatwaves (1st decile tree coverage, 10th decile tree coverage, PWS station average within the GLA, and PWS station average for surrounding rural areas). (a) 15th–17th June, (b) 17th–19th July, and (c) 9th–15th August. Vertical lines show the time and extent of the maximum UHI intensity on each 24 h period (12pm–12pm).

Over the 2015–2022 period, the average UHI intensity was 3.3 °C, greatest during spring (mean of 3.8 °C, max of 7.6 °C) and lowest during winter (mean of 2.9 °C, max of 7.1 °C). An hourly timeseries of temperatures during the 2022 heatwaves (figure 4) shows the average of all PWS within the GLA, the average of those within the top decile (40.2%–99.9% tree canopy coverage), those in the bottom decile (0%–5.4% canopy coverage), and in the rural areas surrounding the GLA administrative boundaries. During these heatwaves, areas in the top decile tree canopy coverage were, on average, 1.2 °C cooler that those in the lowest decile tree canopy coverage. Differences were most apparent at night, when nighttime minimum temperatures were around 2.0 °C lower in the areas with the most tree canopy, while maximum daytime temperatures were on average 0.8 °C lower. The maximum UHI intensities tended to be in the very early morning hours and averaged 4.3 °C across all heatwave days.

**Figure 5.**
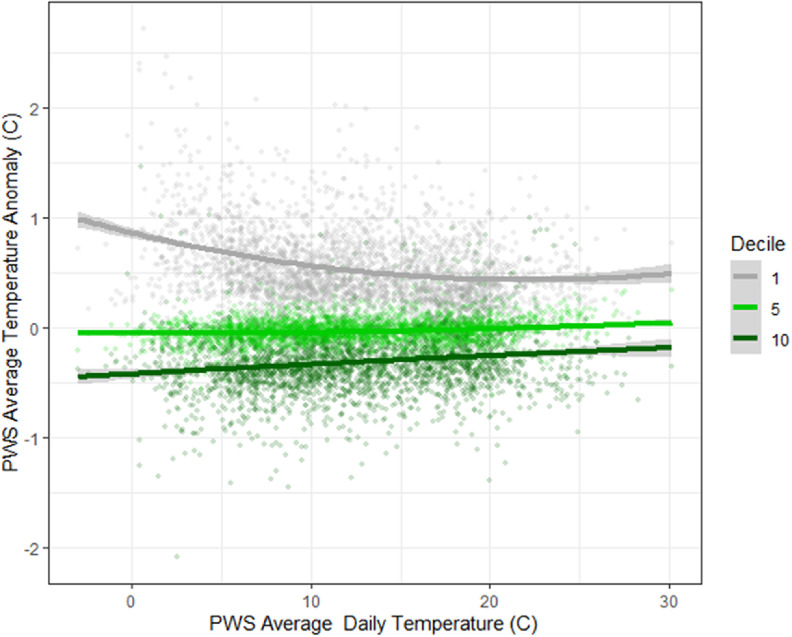
Daily average PWS temperature anomaly (or difference between the average PWS temperature of stations in tree coverage decile, and the average of all PWS stations) by daily average PWS temperature. The bottom (1), median (5) and top (10) decile of tree canopy coverage are shown.

**Figure 6.**
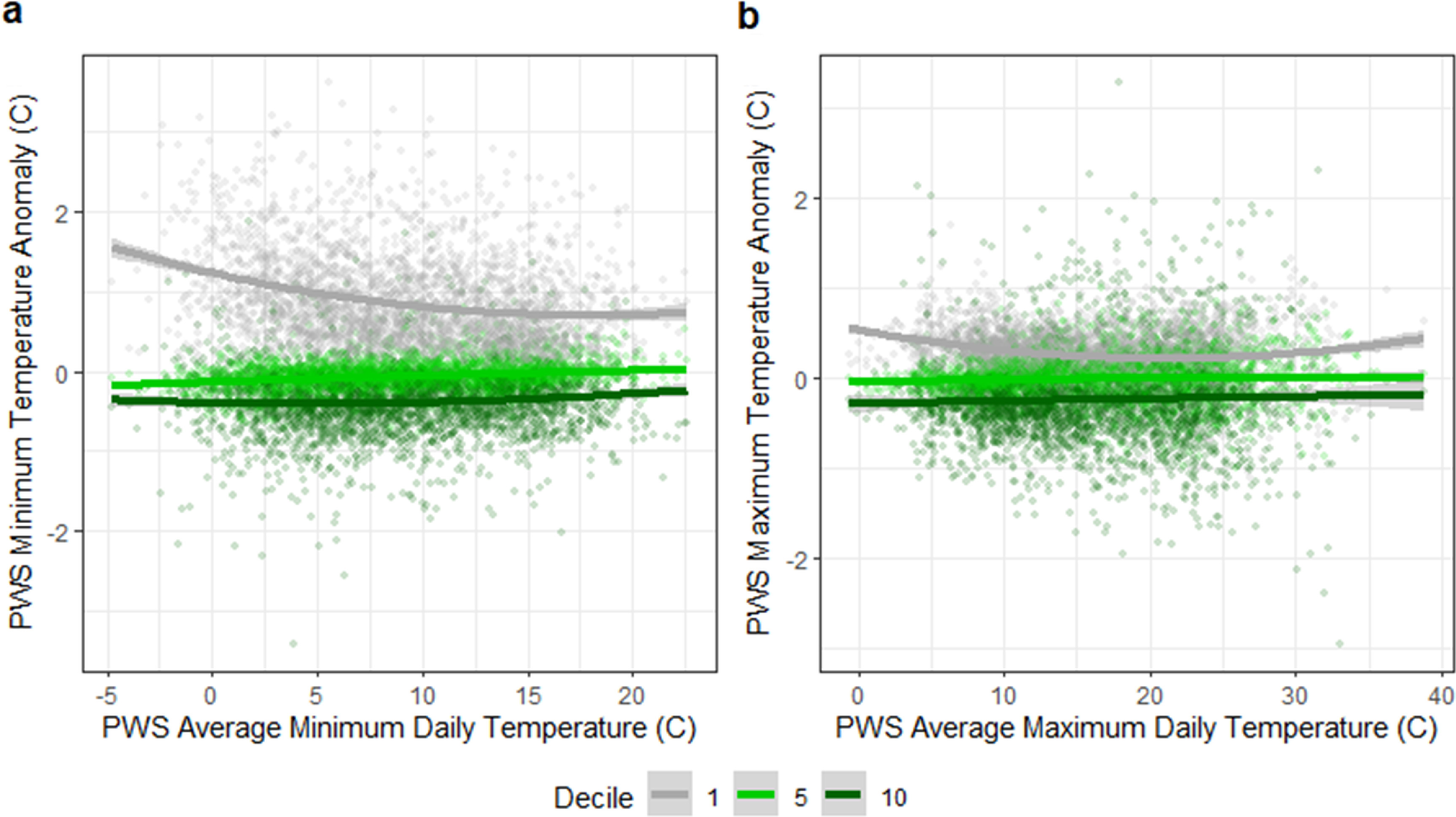
(a) Daily minimum PWS temperature anomaly (or difference between the average minimum PWS temperature of stations in tree coverage decile, and the average minimum of all PWS stations) and average daily minimum PWS temperature, and (b) Daily maximum PWS temperature anomaly (or difference between the average maximum PWS temperature of stations in tree coverage decile, and the average maximum of all PWS stations) and average daily maximum PWS temperature. The bottom (1), median (5) and top (10) decile of tree canopy coverage are shown.

Differences in daily mean, minimum, and maximum temperatures for stations with different levels of tree canopy coverage can be seen over the long-term data (figures 5 and 6). These figures show mean, minimum, and maximum anomalies (or differences between the stations and the average of all stations) as temperatures increase. Temperatures are, on average, 0.8 °C higher in deciles with the least tree coverage compared to the most, or around −0.019 °C (−0.0192 to −0.0187) per % increase in tree canopy. The temperature differences between the areas with the greatest and least amount of tree canopy coverage was greatest at lower temperatures (average minimum difference 1.1 °C), and the least at higher temperatures (average maximum differences 0.6 °C).

### 3.3. Heat exposure and health impact models

As expected, data was noisy and with unexplained variance in the model. The GAM with the 1000 m buffer showed the best performance as judged by chi-squared tests between models (table S1 in the appendix). Negligible amounts of spatial autocorrelation were present in the residuals of this model (an average Morans I of 0.000 41 during summers) compared to the total variation. A cyclical temporal correlation was apparent with peaks during summer; closer examination alongside additional Met Office data indicates that these may be due to exposure to solar radiation. As a result, both spatial and temporal autocorrelation were decided to be unimportant, but we acknowledge that errors may be underestimated because daily samples may not be independent. The final model was able to predict daily temperatures with an *R*2 of 98.3% and a root mean square error (RMSE) of 0.808 for stations and 0.761 for observations on held-out data. Partial dependence plots can be seen in the appendix.

**Figure 7.**
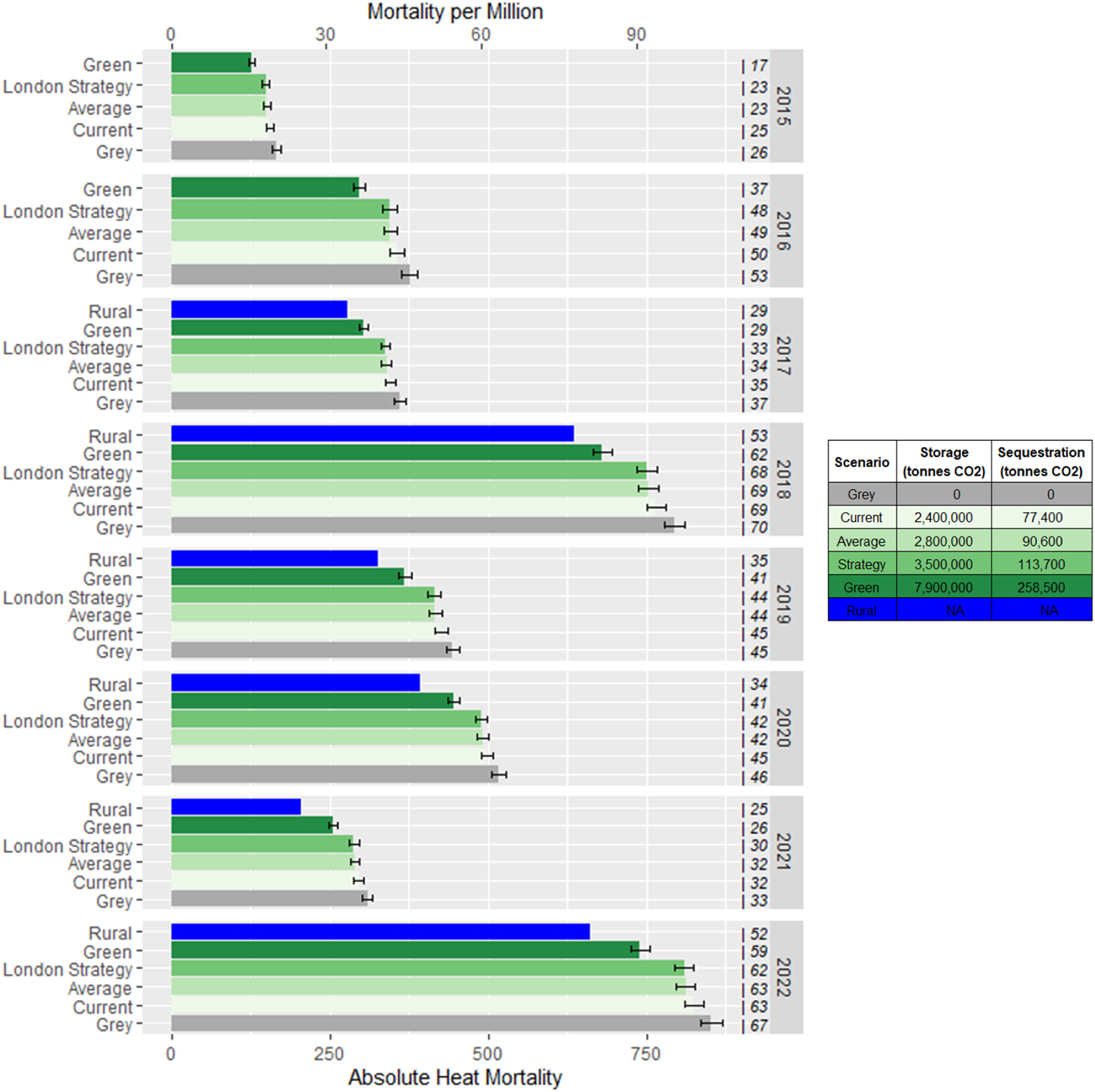
Estimated average yearly heat-associated mortality under the different tree canopy coverage scenarios. The number of days above ${T_h}$ is shown for each year and scenario. Estimates at rural temperatures are excluded for 2015 and 2016 due to low numbers of rural weather stations. Error bars show 95% confidence interval. The estimated storage and sequestration (per annum) for the scenarios is shown in the legend. NB. Estimates after 2020 use daily mortality data that includes covid-related mortality.

**Figure 8.**
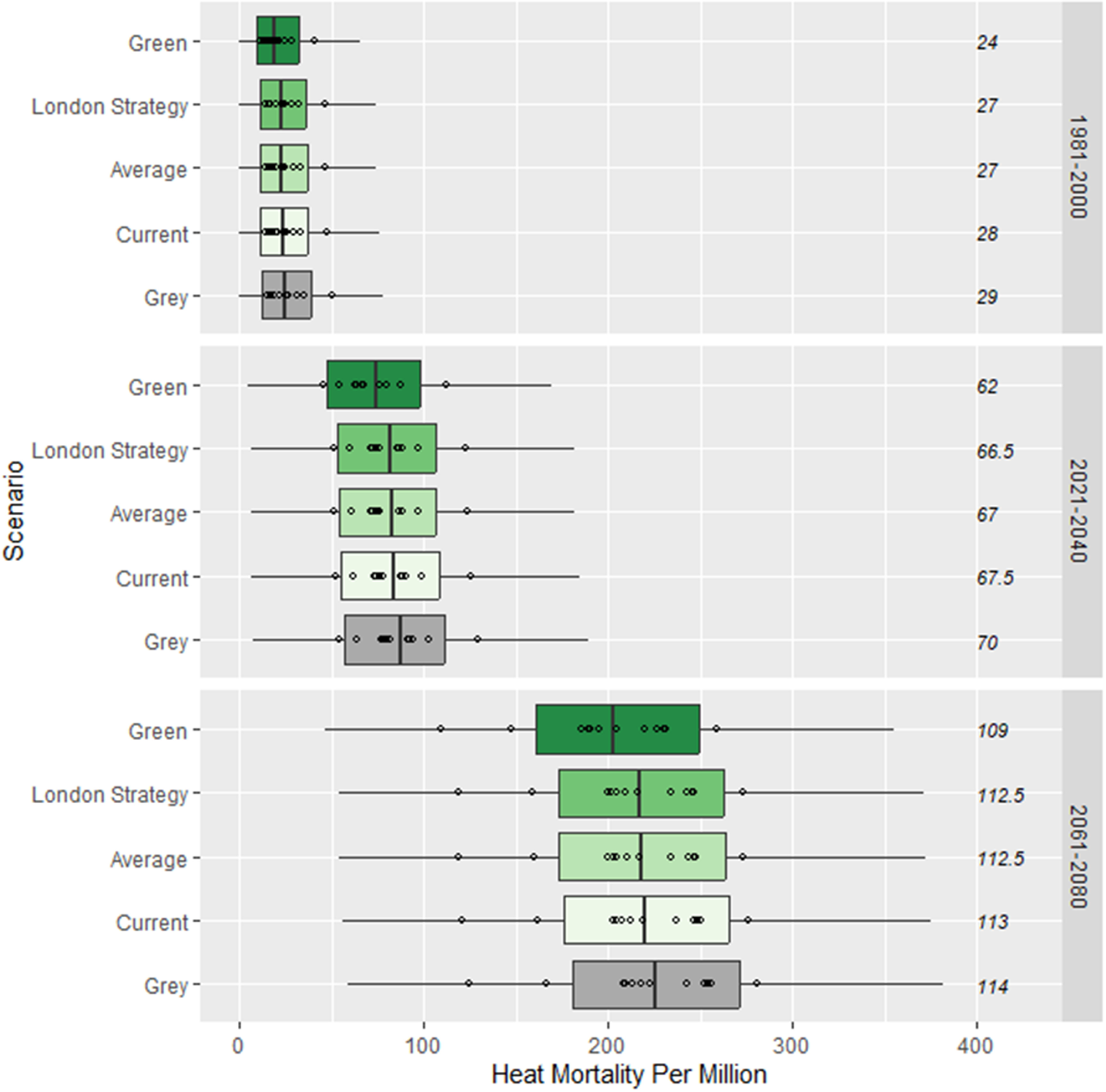
Estimated yearly heat-associated mortality for the different scenarios under projected climates. Distributions represent the range in annual mortality estimates across years and ensemble members. Points indicate the median estimated annual mortality for each ensemble member. The median number of days above ${T_h}$ is shown for each epoch and scenario.

Health impact calculation results can be seen in figures 7 and 8. Between 2015–2022, we estimate that the current tree coverage avoided 153 heat attributable deaths. This represents a 16% reduction of the UHI-related mortality that would have occurred under the ‘grey scenario’. Relative to the current tree canopy coverage, increasing tree coverage to average levels from 2017–2022 would have further reduced UHI mortality by 8%, the London strategy by 10%, and the green scenario by 55%. During the heatwave events of 2022, we estimate that the current tree coverage helped avoid around 16 heat attributable deaths (14% of UHI-associated deaths). This would increase to 23 (21%), 25 (22%), and 67 (61%) under the average, London strategy, and green scenarios, respectively.

Heat mortality is projected to increase around 8-fold by 2061–2080 relative to 1981–2000 as a result of the rising temperatures and the more frequent exceedance of ${T_h}$ under RCP8.5 (figure 8). By 2061–2080, we estimate that the current tree coverage would help avoid around 42 heat-attributable deaths a year. Increased coverage from the London strategy would help avoid an additional 23 deaths a year relative to current levels, and the green scenario 131 deaths a year. The changes also lead to substantial benefits for carbon storage and sequestration, with an additional 1.1 million tonnes of carbon stored and 36 500 tonnes sequestered annually under the London strategy, rising to 5.6 million tonnes stored and 181 000 tonnes sequestered annually under the green scenario.

## 4. Discussion

This paper has used the growing number and density of PWS stations around the GLA to evaluate temperature differences by tree canopy coverage and between urban and surrounding rural areas over a period of seven years. Differences in urban temperatures were observed by tree canopy coverage. During the three heatwaves in 2022, areas with the top 10% of tree canopy coverage were 1.2 °C cooler, on average, then those in the bottom 10% tree canopy coverage. Differences were greatest at night, with temperatures around 2.0 °C lower in the areas with the most tree canopy, versus 0.8 °C lower during the day. This is likely due to the UHI effect, which is more prominent at night, and support prior studies showing the nocturnal cooling effects on greenspace in London [10]. When analysed over the entire timeseries, temperature differences are greatest during cooler weather.

From this data, a GAM model was developed to estimate the influence of tree canopy on temperatures. The model was able to achieve an RMSE of 0.761 and an *R* ^2^ of 98.3% for observations. The highly variable nature of the temperature data meant that there is a high degree of model uncertainty. We have focused here on model simplicity, but further work can help to develop the predictive power further. By including BC and BH as a covariate, we aim to account for the urban heat island effect. Both are unchanged for the scenario predictions, meaning we are comparing hypothetical areas with the same buildings but different tree coverage.

Applying the model to timeseries weather data from different climate scenarios enables changes in heat exposure and subsequent mortality to be predicted. This novel approach enables estimation of near-surface air temperature exposure under different scenarios, building upon past work that has used LST or greenery as an effect modifier. The scale of future mortality under current levels of tree canopy coverage are similar to estimates from other studies [47, 48]. Our estimated impact of trees on reducing mortality lies in between other studies. Iungman *et al* [16], estimated a decrease in UHI-AM of 24.3% in London if tree coverage was increased from 15.5%, to 30%, whereas we estimate that increasing canopy coverage from 21%–31% (London Strategy) would reduce UHI-AM by 10%, comparatively smaller. Our results are higher than those of Choi *et al* [49], whose analysis of heat-mortality and greenspace levels in 425 cities located in 24 countries, estimating that a 20% increase in greenspace could result in a 9% decrease in heat-mortality annually in these cities. Results support epidemiological evidence that trees in London can significantly reduce heat mortality [50].

The effectiveness of the tree canopy at reducing heat exposure and mortality is estimated to slightly decrease as temperatures increase. This is due to the threshold effect of the model, where warm days can be reduced below the heat mortality threshold through adaptations. As temperatures increase, the numbers of days that can be reduced below this threshold decreases, and heat-related mortality rises. This effect could be partially compensated for if populations adapt to heat effects in future and therefore mortality thresholds rise. However, evidence on population adaptation to heat effects is sparse, and time frames for adaptation are uncertain [51].

### 4.1. Limitations and uncertainties

A strength of this study is the large amounts of temperature data with dense coverage. A tradeoff, however, is in the accuracy of the stations compared to official meteorological stations. To minimize errors, we use data cleaning methods to remove outliers, stations with unreliable data, and indoor stations, while all PWS stations are from the same manufacturer and are assumed to have similar instrumental errors. Five official stations are used to supplement coverage, meaning a relatively small number of different instruments are included in the modelling dataset, but the data from these will be more with accurate than those from PWS. Our reference for UHI intensity are stations outside the GLA located in rural areas, and acknowledge that the trends and amplitude of the UHI we report are sensitive to these reference temperatures [52].

By using quantile regression to identify the maximum amount of local tree coverage according to building density, we aimed to estimate a maximal amount of tree canopy that can be added, while the London Strategy and average scenarios present more achievable targets; these scenarios are an advance over prior studies that offered a percent increase in tree coverage unsupported by evidence or policy. Temperatures will also depend on LCZs, but due to the correlation of the natural component with tree coverage we opted to derive from a buildings dataset to reflect the built component. However, tree canopy coverage and buildings are just one factor which contributes to urban temperature differences and may be confounded by other parameters which we have not included here. The GAM model does not include prevailing wind conditions, nor any advective cooling from the river Thames or the sea. The use of hexagons with a relatively small area helps to separate large areas of tree canopy (such as parks) from areas with residential populations. The modelled temperature changes are associative and cross-sectional and do not demonstrate causality, although this is equally a problem with all other studies of this type, e.g [16].

The tree canopy data offers high resolution coverage derived using machine learning processed aerial imagery. The report on the generation of the dataset acknowledges various limitations. These include the data coming from single time point (September 2016) and a time of the year when leaf structure may be breaking down. The model occasionally misclassifies trees as bushes and can miss sections of canopy altogether. However, the model has an accuracy rate of 94% and produces estimates that are at minimum comparable to estimates from traditional survey methods when aggregated across larger areas. Estimates of the stored carbon within London’s trees is based on figures from a 2015 report [42], and may in fact underestimate the amount of carbon stored [53].

The heat-mortality estimates use a relationship between outdoor air temperature and mortality derived for 1996–2013. This model does not include relative humidity or air pollution as confounders, although previous research has not found these to be significant factors for heat mortality in the UK [9, 43]. This heat-mortality relationship uses daily mean temperature, however, there is evidence that nighttime temperatures are particularly important for health outcomes [54]. Given that the greatest effect of tree coverage was seen at night, this could mean that the health benefits of the tree canopy are underestimated. We have not looked at cold. For the future projections, we do not model population social or technological adaptation to heat, such as heatwave plans or use of air conditioning, nor do we model population demographic changes such as population aging. The baseline mortality rate for 2020–2022 will include the effects of Covid-19.

The heat-mortality-relationship used is based on epidemiological analysis of mortality data which partially pre-dates the development of many of the UK heatwave public health measures. Our predicted mortality for the heat periods of the summer of 2022 is both higher than other real-time models and the observed excess mortality [8], likely due to different sources of temperature data, as well as different underlying methods in calculating heat-AM. During these heatwaves, the UK Met Office issued heat-health alert and red EH weather warnings to the public for the first time. It is possible that the warnings and advice from the Met Office and Public Health agencies had a role in reducing the actual mortality below what was expected. The estimated mortality in this study should therefore be interpreted as a theoretical quantification of the role of trees rather than an absolute estimate of heat-related mortality during this period.

### 4.2. Implications

The methods used here can be applied to other locations and with different land covers wherever PWS data are available. While the results of this study can provide an indicative value for similar cities, caution should be applied when generalizing as city-specific characteristics like climate, the amount of potential greening, and the built environment will influence estimates and a recent PWS study shows that increased tree cover in many European cities does not lead to reduced air temperatures [15]. The types of trees, such as their leaf area index, and the albedo of the built environment are also important considerations [55].

In addition to benefits for reducing heat exposure, trees offer a number of benefits, including biodiversity, stormwater management, air pollution removal, and carbon storage and sequestration [42]. Green spaces have been shown to have a positive influence on physical activity, stress, social contacts, and restoration [56], and increased urban street tree density is associated with reduced mental health issues [57]. In the UK, all-cause mortality was found to be 6% lower in the quintile with the most greenspace compared to the quintile with the lowest [58].

Trees also have some disadvantages. Under certain circumstances, trees can risk negatively impacting air quality by preventing dispersion of polluted air in narrow streets for certain wind directions [59, 60], while the pollen produced by certain tree species can exacerbate allergies [61]. Our results show areas with more trees are colder at lower temperatures, with possible increased in space heating energy demand [62] if the necessary energy efficient retrofits are not carried out. Types of trees are likely to be important, with deciduous trees offering beneficial shading during the summer. Future research could compare the estimated costs associated with additional tree coverage with the potential savings provided by reduced health care cost and examine the impacts on indoor temperatures and heating or cooling demand.

## 5. Conclusions

This study examined the differences in air temperatures in London according to tree canopy coverage, focusing in particular on heat. PWS data from 2015–2022 shows that areas with higher tree canopy coverage were associated with reduced levels of heat compared to those with low tree canopy coverage. During the heatwaves of 2022, this corresponded to a lower average difference in maximum daytime temperatures (0.8 °C) and minimum temperatures (2.0 °C). Modelled impacts of different climate and tree canopy coverage scenarios estimates that the existing urban tree canopy coverage in London may have reduced excess heat-related mortality by around 16% during 2015–2022. Increasing the tree canopy to the maximum level, given building density, is estimated to lead to a reduction of 55%. The cooling benefits from trees become even greater under hotter future climates. The results of this study support increasing tree canopy coverage to help mitigate high urban temperatures in the future, with urban greening part of a set of broader public health actions that can help reduce heat-related mortality in the future.

## Data Availability

The data cannot be made publicly available upon publication because they are owned by a third party and the terms of use prevent public distribution. The data that support the findings of this study are available upon reasonable request from the authors.
